# Supplementary material for: Determinants of Vaccination Uptake in Risk Populations: A Comprehensive Literature Review
Source: Vaccines (Basel). 2020 Aug 27;8(3):480. doi: 10.3390/vaccines8030480 (PMC7563537; doi:10.3390/vaccines8030480)
Supplement: Supplementary file 1 [file vaccines-08-00480-s001.zip › Supplementary Table S2. Quality assessment.pdf]

**AXIS items scoring**	Balaban, 2013	Barasheed, 2014	Duffy, 2013	Frew, 2017
Were the aims/objectives of the study clear?	1	1	1	1
Was the study design appropriate for the stated aim(s)?	0	1	1	1
Was the sample size justified?	0	1	1	0
Was the target/reference population clearly defined? (Is it clear who the research was about?)	0	1	1	1
Was the sample frame taken from an appropriate population base so that it closely represented the target/reference population under investigation?	x	1	1	1
Was the selection process likely to select subjects/participants that were representative of the target/reference population under investigation?	x	1	1	1
Were measures undertaken to address and categorise non-responders?	x	0	0	0
Were the risk factor and outcome variables measured appropriate to the aims of the study?	1	1	1	1
Were the risk factor and outcome variables measured correctly using instruments/measurements that had been trialled, piloted or published previously?	x	1	0	0
Is it clear what was used to determined statistical significance and/or precision estimates? (eg, p values, CIs)	1	1	1	1
Were the methods (including statistical methods) sufficiently described to enable them to be repeated?	0	0	0	0
Were the basic data adequately described?	0	1	1	1
**Does the response rate raise concerns about non-response bias? (1 = no concerns)**	x	1	0	1
If appropriate, was information about non-responders described?	x	0	0	0
Were the results internally consistent?	1	1	1	1
Were the results for the analyses described in the methods, presented?	x	0	1	1
Were the authors’ discussions and conclusions justified by the results?	1	1	1	1
Were the limitations of the study discussed?	0	1	1	1
**Were there any funding sources or conflicts of interest that may affect the authors’ interpretation of the results? (1 = no conflicts/funding)**	1	0	1	1
Was ethical approval or consent of participants attained?	x	1	0	1
**TOTAL**	6	15	14	15

Goodman, 2014	Herbinger, 2011	Heywood, 2016	Igreja, 2019	Lammert, 2016	Paudel, 2017	Pavli, 2019	Pfeil, 2010	Selcuk, 2016	Tan, 2017	Tashani, 2016	Wiemken, 2015	Yanni, 2010	Akin, 2016	Althoff, 2010	Battistella, 2019	Chehab, 2018	Chin-Yee, 2011	Gagneux-Brunon, 2019	Haroon, 2011	Harrison, 2017	Harrison, 2018	Lachenal, 2010	Loubet, 2015	Loubet, 2018	Malhi, 2015	Miller, 2018	Mouthon, 2010	Narula, 2012	Nguyen, 2017	Poeppl, 2015	Price, 2019	Restivo, 2017	Ruiz-Cuesta, 2016	Sadlier, 2015	Sandler, 2016	Savage, 2011	Struijk, 2015	Teich, 2011
1	1	1	1	1	1	1	1	0	1	1	1	1	1	1	1	1	1	1	1	1	1	0	1	1	1	1	1	1	1	1	1	1	1	1	1	1	1	1
1	0	1	1	1	1	0	1	1	1	1	1	1	1	1	1	1	1	1	1	1	1	1	1	1	1	1	1	1	1	1	1	1	1	1	1	1	1	1
0	1	1	0	1	1	1	1	0	1	1	0	1	0	1	1	1	0	1	1	1	1	1	1	1	1	0	0	1	1	1	1	0	0	0	0	0	0	0
1	1	1	1	1	1	1	0	1	1	1	1	1	1	1	0	1	1	0	1	1	0	1	1	1	1	1	1	0	1	0	1	1	1	1	1	1	1	1
1	0	1	0	1	1	1	1	1	1	1	1	1	1	1	1	1	1	0	1	1	1	1	0	0	1	1	1	1	1	1	1	1	1	1	1	1	1	1
0	0	0	0	0	1	1	1	0	1	0	1	1	1	1	1	1	1	x	1	1	1	0	0	0	1	1	0	1	1	0	1	1	1	1	1	0	1	1
0	0	0	0	x	0	0	0	0	0	0	0	0	0	0	0	0	0	0	x	0	0	0	0	0	0	0	0	1	0	0	0	0	0	0	1	0	1	1
1	0	1	1	x	1	1	1	1	1	1	1	1	1	1	1	1	1	1	1	1	1	1	1	0	1	1	1	0	1	0	0	1	1	0	1	1	1	1
0	0	0	0	0	0	0	0	1	0	0	1	0	0	0	1	1	0	0	0	0	0	1	0	0	0	1	0	0	0	0	1	0	0	1	1	1	1	1
1	1	x	x	1	1	1	1	1	1	0	1	1	1	0	1	1	0	0	0	1	1	0	1	1	1	1	1	1	1	1	0	1	1	1	1	1	1	1
0	1	0	0	1	0	1	1	1	1	1	1	0	0	0	1	0	1	0	0	0	0	0	0	1	0	1	1	0	1	1	1	0	0	0	1	1	1	1
0	1	0	1	1	1	1	1	1	1	1	1	1	1	1	1	1	1	0	1	0	0	1	1	1	1	1	1	1	1	1	0	1	1	1	1	1	1	1
0	0	0	0	1	0	0	1	0	x	1	0	1	1	1	0	1	1	1	1	x	x	1	0	x	0	0	x	1	1	0	0	x	0	1	0	0	1	1
x	0	0	0	x	0	0	0	0	x	0	0	1	0	0	0	0	0	0	x	x	x	0	0	x	0	0	x	1	0	0	0	x	0	0	0	0	1	1
1	1	1	1	1	1	1	0	0	1	1	1	1	1	1	1	1	1	1	1	1	1	1	1	1	1	1	1	1	1	1	1	1	1	1	1	1	1	1
1	1	1	x	1	0	1	1	1	1	1	1	1	1	1	1	1	1	0	1	1	1	0	1	1	1	1	1	1	1	1	1	1	1	0	1	1	1	1
1	0	1	1	1	1	1	1	1	1	1	1	1	1	1	1	1	1	1	1	1	1	1	1	1	1	1	1	1	1	0	1	1	1	1	1	1	1	1
1	1	1	0	1	1	1	1	1	1	1	1	1	0	0	1	0	0	0	0	0	1	0	1	1	1	1	0	1	1	0	1	1	1	0	1	1	1	1
1	0	0	1	1	0	0	1	1	1	0	0	1	1	1	1	1	1	1	1	1	1	1	0	1	1	1	1	x	x	1	1	1	0	1	1	1	1	1
1	1	1	0	1	1	1	1	1	1	1	1	1	1	1	1	1	1	x	1	1	1	1	1	1	1	1	1	1	1	1	1	1	1	0	0	0	1	0
12	10	11	8	15	13	14	15	13	16	14	15	17	14	14	16	16	14	8	14	13	13	12	12	13	15	16	13	15	16	11	14	14	13	12	16	14	19	18

Urun, 2013	Waszczuk, 2018	Wilckens, 2011	Akan, 2016	Asma, 2016	Boey, 2018	Bonaccorsi, 2015	Castilla, 2013	Ciftci, 2018	Costantino, 2019	Dedoukou, 2010	deSante, 2010	Dominiquez, 2013	Durando, 2016	Ehrenstein, 2010	Giese, 2016	Gramegna, 2018	Gutknecht, 2016	Hagemeister, 2018	Harrison, 2016	Hopman, 2010	Hulo, 2017	Johansen, 2012	Kalemaki, 2020	Karlsson, 2019	Kisic-Tepavcevic, 2017	Lehmann, 2015	Maridor, 2017	Napolitano, 2019	Nowrouzi, 2014	Pielak, 2010	Prematunge, 2014	Quan, 2012	Rabensteiner, 2018	Real, 2013	Rebmann, 2012	Scatigna, 2017	Surtees, 2018	Taddei, 2014
1	1	1	1	1	1	1	1	1	1	1	1	1	1	1	1	1	1	1	1	1	1	1	1	1	1	1	1	1	1	1	1	1	1	1	1	1	1	1
1	1	1	1	1	1	1	1	1	1	1	1	1	1	1	1	1	1	1	1	1	1	1	1	1	1	1	1	1	1	1	1	1	1	1	1	1	1	1
0	0	0	0	0	1	0	1	0	0	0	0	1	0	0	1	0	0	0	0	0	0	0	0	0	0	0	0	1	0	0	0	0	0	0	0	0	0	0
1	1	1	1	1	1	0	1	1	1	1	1	1	1	1	1	1	1	1	1	1	1	1	1	1	1	1	1	1	1	1	1	1	1	1	1	1	1	1
1	1	1	1	0	1	1	1	1	1	1	0	1	1	1	0	0	0	1	1	0	1	0	1	0	1	1	1	1	1	1	1	1	1	1	1	1	1	1
1	1	1	1	0	1	0	1	1	1	0	1	1	1	1	0	0	0	1	x	1	1	1	1	1	1	1	1	1	1	1	1	0	1	0	1	1	1	0
0	0	1	1	0	1	0	1	0	1	0	0	0	0	0	0	0	0	0	0	0	0	0	1	0	0	0	0	0	0	0	0	0	0	0	0	0	0	0
1	1	1	1	1	1	1	1	1	1	1	1	1	1	1	1	1	1	1	1	1	1	1	1	1	1	1	1	1	1	1	1	1	1	1	1	1	1	1
1	1	1	1	1	1	1	1	1	0	0	0	1	1	0	0	0	0	0	0	1	0	1	0	1	1	1	1	1	1	1	1	0	1	1	1	0	0	x
1	1	0	1	1	1	1	1	1	1	1	1	1	1	1	1	1	x	1	1	1	1	1	1	1	1	1	0	1	1	1	x	1	1	1	1	1	1	1
0	1	0	1	0	1	1	1	1	0	0	0	0	0	1	0	0	0	1	0	0	0	0	0	1	0	0	0	0	1	1	0	0	0	0	0	0	1	0
1	1	1	1	1	1	1	1	1	1	1	1	1	1	1	1	1	1	0	1	1	1	1	1	1	1	1	1	1	1	1	1	1	1	1	1	1	1	1
1	0	1	0	1	1	0	0	0	1	0	0	0	0	0	0	0	0	0	0	0	0	1	1	0	1	0	0	1	0	0	0	1	0	0	0	0	1	0
0	0	1	0	0	1	0	1	0	0	0	0	0	0	0	0	0	0	0	0	0	0	0	1	0	0	0	0	0	0	0	0	0	0	0	0	0	1	0
1	1	1	1	1	1	1	1	1	1	1	1	1	1	1	1	1	1	x	1	1	1	0	1	1	1	1	1	1	1	1	1	1	1	1	1	1	1	1
1	1	1	1	1	1	1	1	1	1	1	1	1	1	1	1	1	1	1	1	1	1	1	1	1	1	1	1	1	1	1	1	1	1	1	1	1	1	1
1	1	1	1	1	1	1	1	1	1	1	1	1	1	1	1	1	1	1	1	1	1	1	1	1	1	1	1	1	1	1	1	1	1	1	1	1	1	1
0	1	1	1	1	1	1	1	1	1	0	1	1	1	1	1	1	0	1	0	1	0	1	1	1	1	1	1	1	1		1	0	1	1	1	1	1	1
1	1	1	1	1	1	1	1	1	1	x	x	x	1	1	1	x	1	1	1	1	1	x	1	1	1	1	x	1	x	1	1	1	1	x	1	1	1	1
0	1	0	1	0	1	0	0	0	x	x	1	1	1	x	1	x	x	1	1	x	1	1	1	1	1	1	1	1	1	1	1	x	1	1	1	1	1	1
14	16	16	17	13	20	13	18	15	15	10	12	15	15	14	13	10	9	13	12	13	13	13	17	15	16	15	13	17	15	15	14	12	15	13	15	14	17	13

Tanguy, 2018	Vallée-Tourange, 2018	Verger, 2016	Virseda, 2010	Wicker, 2010	Wilson, 2019	Wilson, 2020	Zhang, 2011 & 2012
1	1	1	1	1	1	1	1
1	1	1	1	1	1	1	1
0	0	0	1	0	0	0	0
1	1	1	1	1	0	1	1
1	1	1	1	1	0	0	0
1	1	1	1	1	1	1	1
0	0	1	0	0	1	0	0
1	1	1	1	1	1	1	1
0	0	1	0	0	1	1	1
0	1	1	1	1	1	1	1
0	1	0	0	0	0	0	0
1	1	1	1	1	1	1	1
0	0	0	0	0	0	0	0
0	0	1	0	0	0	0	0
1	1	1	1	1	1	1	1
1	1	1	1	1	1	1	1
1	1	1	1	1	1	1	1
0	1	1	1	1	1	1	1
0	0	1	x	1	1	1	0
1	1	1	1	1	x	x	1
11	14	17	14	14	13	13	13
