# Supplementary material for: Determinants of Vaccination Uptake in Risk Populations: A Comprehensive Literature Review
Source: Vaccines (Basel). 2020 Aug 27;8(3):480. doi: 10.3390/vaccines8030480 (PMC7563537; doi:10.3390/vaccines8030480)
Supplement: Supplementary file 1 [file vaccines-08-00480-s001.zip › Supplementary File S1. Search strings databases.pdf]

Supplementary File S1. Search strings for databases

Database searched	via	Years of coverage	References	After de-duplication
Embase	Embase.com	1971 - Present	1306	1293
Medline ALL	Ovid	1946 - Present	467	114
CINAHL	EBSCO	1982 - Present	29	1
Web of Science Core Collection	Web of Knowledge	1975 - Present	99	14
PsycINFO	Ovid	1806 - Present	233	187
ERIC	Ovid	1965 - Present	41	37
SocINDEX	EBSCO	1895 - Present	52	28
**Total**			**2227**	**1674**

References before 2010: 403 (exclusion folder in EndNote)
References since 2010: 1271 (unfiled in EndNote)

Embase.com

(('vaccination coverage'/de OR (((vaccin* OR immuniz* OR immunis*) NEAR/3 (cover* OR uptake* OR adherence* OR compliance* OR hesitan* OR behavior* OR behaviour* OR decision* OR status* OR rate* OR acceptanc* OR refus*))):ab,ti,kw) AND ('behavior'/exp OR (((behavior* OR behaviour* OR habit* OR practice* OR attitude* OR ASE OR trans-theor* OR health-belief* OR social-cognitive* OR precautions-adoption-process* OR integrated-change* OR i-change* OR theor*) NEAR/3 (change* OR modif* OR alter* OR model* OR framework* OR intervention* OR theor*)) OR fishbein OR ajzen OR prochaska OR diclemente OR hochbaum OR kegels OR rosenstock OR bandura OR Weinstein OR de- vries):ab,ti,kw) AND ('immunocompromised patient'/de OR 'autoimmune disease'/exp OR 'inflammatory bowel disease'/exp OR 'Human immunodeficiency virus infected patient'/exp OR 'graft recipient'/de OR 'cancer patient'/exp OR (immunocompromi* OR compromi* OR immunosuppress* OR autoimmune* OR auto-immune* OR arthritis* OR rheuma* OR psoriasis* OR IBD OR SLE OR lupus* OR systemic-scleros* OR inflammatory-bowel* OR crohn* OR colitis* OR ((HIV OR immunodeficiency OR cancer*) NEAR/3 (patient* OR adult* OR survivor* OR person*)) OR ((transplant* OR graft*) NEAR/3 (recipient* OR host* OR stem-cell* OR organ*))):ab,ti,kw)) **OR** ((('vaccination coverage'/de AND 'health care personnel'/exp) OR (((vaccin* OR immuniz* OR immunis*) NEAR/3 (cover* OR uptake* OR adherence* OR compliance* OR hesitan* OR behavior* OR behaviour* OR decision* OR status* OR rate* OR acceptanc* OR refus*) NEAR/3 (clinician* OR nurs* OR physician* OR specialist* OR doctor* OR personnel OR provider* OR health-care-worker* OR HCWs OR HCW OR professional* OR practitioner*))):ab,ti,kw) AND ('behavior'/exp OR (((behavior* OR behaviour* OR ASE OR trans-theor* OR health-belief* OR

social- cognitive* OR precautions-adoption-process* OR integrated-change* OR i-change* OR theor*) NEAR/3 (change* OR modif* OR alter* OR model* OR framework* OR intervention* OR theor*)) OR fishbein OR ajzen OR prochaska OR diclemente OR hochbaum OR kegels OR rosenstock OR bandura OR Weinstein OR de-vries):ab,ti,kw)) **OR** (('vaccination coverage'/de OR (((vaccin* OR immuniz* OR immunis* OR guideline*) NEAR/3 (cover* OR uptake* OR adherence* OR compliance* OR hesitan* OR behavior* OR behaviour* OR decision* OR status* OR rate* OR acceptanc* OR refus*))):ab,ti,kw) AND ('behavior'/exp

OR (((behavior* OR behaviour* OR habit* OR practice* OR attitude* OR ASE OR trans-theor* OR health- belief* OR social-cognitive* OR precautions-adoption-process* OR integrated-change* OR i-change* OR theor*) NEAR/3 (change* OR modif* OR alter* OR model* OR framework* OR intervention* OR theor*)) OR fishbein OR ajzen OR prochaska OR diclemente OR hochbaum OR kegels OR rosenstock OR bandura OR Weinstein OR de-vries):ab,ti,kw) AND ('travel'/de OR 'tourism'/de OR (travel* OR VFR OR visiting- friends-and-relatives* OR expat* OR emigrant* OR pilgrim* OR touris*):ab,ti,kw)) AND [english]/lim NOT ([Conference Abstract]/lim AND [1800-2017]/py)

Medline (Ovid)

((Vaccination Coverage/ OR (((vaccin* OR immuniz* OR immunis*) ADJ3 (cover* OR uptake* OR adherence* OR compliance* OR hesitan* OR behavior* OR behaviour* OR decision* OR status* OR rate* OR acceptanc* OR refus*))).ab,ti,kf.) AND (exp Behavior/ OR (((behavior* OR behaviour* OR habit* OR practice* OR attitude* OR ASE OR trans-theor* OR health-belief* OR social-cognitive* OR precautions-adoption-process* OR integrated-change* OR i-change* OR theor*) ADJ3 (change* OR modif* OR alter* OR model* OR framework* OR intervention* OR theor*)) OR fishbein OR ajzen OR prochaska OR diclemente OR hochbaum OR kegels OR rosenstock OR bandura OR Weinstein OR de- vries).ab,ti,kf.) AND (Immunocompromised Host/ OR exp Autoimmune Diseases/ OR exp Inflammatory Bowel Diseases/ OR Transplant Recipients/ OR (immunocompromi* OR compromi* OR immunosuppress* OR autoimmune* OR auto-immune* OR arthritis* OR rheuma* OR psoriasis* OR IBD OR SLE OR lupus* OR systemic-scleros* OR inflammatory-bowel* OR crohn* OR colitis* OR ((HIV OR immunodeficiency OR cancer*) ADJ3 (patient* OR adult* OR survivor* OR person*)) OR ((transplant* OR graft*) ADJ3 (recipient* OR host* OR stem-cell* OR organ*))).ab,ti,kf.)) OR (((Vaccination Coverage/ AND exp Health Personnel/) OR (((vaccin* OR immuniz* OR immunis*) ADJ3 (cover* OR uptake* OR adherence* OR compliance* OR hesitan* OR behavior* OR behaviour* OR decision* OR status* OR rate* OR acceptanc* OR refus*) ADJ3 (clinician* OR nurs* OR physician* OR specialist* OR doctor* OR personnel OR provider* OR health-care-worker* OR HCWs OR HCW OR professional* OR practitioner*))).ab,ti,kf.) AND (exp Behavior/ OR (((behavior* OR behaviour* OR ASE OR trans-theor* OR health-belief* OR social-cognitive* OR precautions-adoption-process* OR integrated-change* OR i- change* OR theor*) ADJ3 (change* OR modif* OR alter* OR model* OR framework* OR intervention* OR theor*)) OR fishbein OR ajzen OR prochaska OR diclemente OR hochbaum OR kegels OR rosenstock OR bandura OR Weinstein OR de-vries).ab,ti,kf.)) OR ((Vaccination Coverage/ OR (((vaccin* OR immuniz* OR immunis* OR guideline*) ADJ3 (cover* OR uptake* OR adherence* OR compliance* OR hesitan* OR behavior* OR behaviour* OR decision* OR status* OR rate* OR acceptanc* OR refus*))).ab,ti,kf.) AND (exp Behavior/ OR (((behavior* OR behaviour* OR habit* OR practice* OR attitude* OR ASE OR trans- theor* OR health-belief* OR social-cognitive* OR precautions-adoption-process* OR integrated-change* OR i-change* OR theor*) ADJ3 (change*

OR modif* OR alter* OR model* OR framework* OR intervention* OR theor*)) OR fishbein OR ajzen OR prochaska OR diclemente OR hochbaum OR kegels OR rosenstock OR bandura OR Weinstein OR de-vries).ab,ti,kf.) AND (Travel/ OR (travel* OR VFR OR visiting-friends-and-relatives* OR expat* OR emigrant* OR pilgrim* OR touris*).ab,ti,kf.)) AND english.lg.

Web of Science

TS=((((((vaccin* OR immuniz* OR immunis*) NEAR/2 (cover* OR uptake* OR adherence* OR compliance* OR hesitan* OR behavior* OR behaviour* OR decision* OR status* OR rate* OR acceptanc* OR refus*)))) AND ((((behavior* OR behaviour* OR habit* OR practice* OR attitude* OR ASE OR trans- theor* OR health-belief* OR social-cognitive* OR precautions-adoption-process* OR integrated-change* OR i-change* OR theor*) NEAR/2 (change* OR modif* OR alter* OR model* OR framework* OR intervention* OR theor*)) OR fishbein OR ajzen OR prochaska OR diclemente OR hochbaum OR kegels OR rosenstock OR bandura OR Weinstein OR de-vries)) AND ((immunocompromi* OR compromi* OR immunosuppress* OR autoimmune* OR auto-immune* OR arthritis* OR rheuma* OR psoriasis* OR IBD OR SLE OR lupus* OR systemic-scleros* OR inflammatory-bowel* OR crohn* OR colitis* OR ((HIV OR immunodeficiency OR cancer*) NEAR/2 (patient* OR adult* OR survivor* OR person*)) OR ((transplant* OR graft*) NEAR/2 (recipient* OR host* OR stem-cell* OR organ*))))) OR (((((vaccin* OR immuniz* OR immunis*) NEAR/2 (cover* OR uptake* OR adherence* OR compliance* OR hesitan* OR behavior* OR behaviour* OR decision* OR status* OR rate* OR acceptanc* OR refus*) NEAR/2 (clinician* OR nurs* OR physician* OR specialist* OR doctor* OR personnel OR provider* OR health-care-worker* OR HCWs OR HCW OR professional* OR practitioner*)))) AND ((((behavior* OR behaviour* OR ASE OR trans-theor* OR health-belief* OR social-cognitive* OR precautions-adoption-process* OR integrated-change* OR i- change* OR theor*) NEAR/2 (change* OR modif* OR alter* OR model* OR framework* OR intervention* OR theor*)) OR fishbein OR ajzen OR prochaska OR diclemente OR hochbaum OR kegels OR rosenstock OR bandura OR Weinstein OR de-vries))) OR (((((vaccin* OR immuniz* OR immunis* OR guideline*) NEAR/2 (cover* OR uptake* OR adherence* OR compliance* OR hesitan* OR behavior* OR behaviour* OR decision* OR status* OR rate* OR acceptanc* OR refus*)))) AND ((((behavior* OR behaviour* OR habit* OR practice* OR attitude* OR ASE OR trans-theor* OR health-belief* OR social- cognitive* OR precautions-adoption-process* OR integrated-change* OR i-change* OR theor*) NEAR/2 (change* OR modif* OR alter* OR model* OR framework* OR intervention* OR theor*)) OR fishbein OR ajzen OR prochaska OR diclemente OR hochbaum OR kegels OR rosenstock OR bandura OR Weinstein OR de-vries)) AND ((travel* OR VFR OR visiting-friends-and-relatives* OR expat* OR emigrant* ORpilgrim* OR touris*)))) AND DT=(Article OR Review) AND LA=English

PsycINFO (Ovid)

((Immunization/ OR (((vaccin* OR immuniz* OR immunis*) ADJ3 (cover* OR uptake* OR adherence* OR compliance* OR hesitan* OR behavior* OR behaviour* OR decision* OR status* OR rate* ORacceptanc* OR refus*))).ab,ti.) AND (exp Behavior/ OR (((behavior* OR behaviour* OR habit* OR practice* OR attitude* OR ASE OR trans-theor* OR health-belief* OR social-

cognitive* OR precautions-adoption- process* OR integrated-change* OR i-change* OR theor*) ADJ3 (change* OR modif* OR alter* OR model* OR framework* OR intervention* OR theor*)) OR fishbein OR ajzen OR prochaska OR diclemente OR hochbaum OR kegels OR rosenstock OR bandura OR Weinstein OR de-vries).ab,ti.) AND

(Immunodepression/ OR exp Arthritis/ OR exp Colitis/ OR (immunocompromi* OR compromi* OR immunosuppress* OR autoimmune* OR auto-immune* OR arthritis* OR rheuma* OR psoriasis* OR IBD OR SLE OR lupus* OR systemic-scleros* OR inflammatory-bowel* OR crohn* OR colitis* OR ((HIV OR immunodeficiency OR cancer*) ADJ3 (patient* OR adult* OR survivor* OR person*)) OR ((transplant* OR graft*) ADJ3 (recipient* OR host* OR stem-cell* OR organ*))).ab,ti.)) OR (((Immunization/ AND exp Health Personnel/) OR (((vaccin* OR immuniz* OR immunis*) ADJ3 (cover* OR uptake* OR adherence* OR compliance* OR hesitan* OR behavior* OR behaviour* OR decision* OR status* OR rate* OR acceptanc* OR refus*) ADJ3 (clinician* OR nurs* OR physician* OR specialist* OR doctor* OR personnel OR provider* OR health-care-worker* OR HCWs OR HCW OR professional* OR practitioner*))).ab,ti.) AND (exp Behavior/ OR (((behavior* OR behaviour* OR ASE OR trans-theor* OR health-belief* OR social-cognitive* OR precautions-adoption-process* OR integrated-change* OR i-change* OR theor*) ADJ3 (change* OR modif* OR alter* OR model* OR framework* OR intervention* OR theor*)) OR fishbein OR ajzen OR prochaska OR diclemente OR hochbaum OR kegels OR rosenstock OR bandura OR Weinstein OR de-vries).ab,ti.)) OR ((Immunization/ OR (((vaccin* OR immuniz* OR immunis* OR guideline*) ADJ3 (cover* OR uptake* OR adherence* OR compliance* OR hesitan* OR behavior* OR behaviour* OR decision* OR status* OR rate* OR acceptanc* OR refus*))).ab,ti.) AND (exp Behavior/ OR (((behavior* OR behaviour* OR habit* OR practice* OR attitude* OR ASE OR trans-theor* OR health-belief* OR social-cognitive* OR precautions-adoption-process* OR integrated-change* OR i-change* OR theor*) ADJ3 (change* OR modif* OR alter* OR model* OR framework* OR intervention* OR theor*)) OR fishbein OR ajzen OR prochaska OR diclemente OR hochbaum OR kegels OR rosenstock OR bandura OR Weinstein OR de-vries).ab,ti.) AND (Traveling/ OR Tourism/ OR (travel* OR VFR OR visiting-friends-and- relatives* OR expat* OR emigrant* OR pilgrim* OR touris*).ab,ti.)) AND English.lg.

ERIC (Ovid)

((Immunization Programs/ OR (((vaccin* OR immuniz* OR immunis*) ADJ3 (cover* OR uptake* OR adherence* OR compliance* OR hesitan* OR behavior* OR behaviour* OR decision* OR status* OR rate* OR acceptanc* OR refus*))).ab,ti.) AND (exp Behavior/ OR (((behavior* OR behaviour* OR habit* OR practice* OR attitude* OR ASE OR trans-theor* OR health-belief* OR social-cognitive* OR precautions-adoption-process* OR integrated-change* OR i-change* OR theor*) ADJ3 (change* OR modif* OR alter* OR model* OR framework* OR intervention* OR theor*)) OR fishbein OR ajzen OR prochaska OR diclemente OR hochbaum OR kegels OR rosenstock OR bandura OR Weinstein OR de- vries).ab,ti.) AND ((immunocompromi* OR compromi* OR immunosuppress* OR autoimmune* OR auto-immune* OR arthritis* OR rheuma* OR psoriasis* OR IBD OR SLE OR lupus* OR systemic-scleros* OR inflammatory-bowel* OR crohn* OR colitis* OR ((HIV OR immunodeficiency OR cancer*) ADJ3 (patient* OR adult* OR survivor* OR person*)) OR ((transplant* OR graft*) ADJ3 (recipient* OR host* OR stem-cell* OR organ*))).ab,ti.)) OR (((Immunization Programs/ AND exp Health Personnel/) OR (((vaccin* OR immuniz* OR immunis*) ADJ3 (cover* OR

uptake* OR adherence* OR compliance* OR
hesitan* OR behavior* OR behaviour* OR decision* OR status* OR rate* OR acceptanc* OR refus*) ADJ3 (clinician* OR nurs* OR physician* OR specialist* OR doctor* OR personnel OR provider* OR health- care-worker* OR HCWs OR HCW OR professional* OR practitioner*))).ab,ti.) AND (exp Behavior/ OR (((behavior* OR behaviour* OR ASE OR trans-theor* OR health-belief* OR social-cognitive* OR

precautions-adoption-process* OR integrated-change* OR i-change* OR theor*) ADJ3 (change* OR modif* OR alter* OR model* OR framework* OR intervention* OR theor*)) OR fishbein OR ajzen OR prochaska OR diclemente OR hochbaum OR kegels OR rosenstock OR bandura OR Weinstein OR de- vries).ab,ti.)) OR ((Immunization Programs/ OR (((vaccin* OR immuniz* OR immunis* OR guideline*) ADJ3 (cover* OR uptake* OR adherence* OR compliance* OR hesitan* OR behavior* OR behaviour* OR decision* OR status* OR rate* OR acceptanc* OR refus*))).ab,ti.) AND (exp Behavior/ OR (((behavior* OR behaviour* OR habit* OR practice* OR attitude* OR ASE OR trans-theor* OR health-belief* OR social-cognitive* OR precautions-adoption-process* OR integrated-change* OR i-change* OR theor*) ADJ3 (change* OR modif* OR alter* OR model* OR framework* OR intervention* OR theor*)) OR fishbein OR ajzen OR prochaska OR diclemente OR hochbaum OR kegels OR rosenstock OR bandura OR Weinstein OR de-vries).ab,ti.) AND (Travel/ OR Tourism/ OR (travel* OR VFR OR visiting-friends-and-relatives* OR expat* OR emigrant* OR pilgrim* OR touris*).ab,ti.)) AND english.lg.

CINAHL EBSCO

((TI (((vaccin* OR immuniz* OR immunis*) N2 (cover* OR uptake* OR adherence* OR compliance* OR hesitan* OR behavior* OR behaviour* OR decision* OR status* OR rate* OR acceptanc* OR refus*))) OR **AB** (((vaccin* OR immuniz* OR immunis*) N2 (cover* OR uptake* OR adherence* OR compliance* OR hesitan* OR behavior* OR behaviour* OR decision* OR status* OR rate* OR acceptanc* OR refus*)))**) AND (TI** (((behavior* OR behaviour* OR habit* OR practice* OR attitude* OR ASE OR trans-theor* OR health-belief* OR social-cognitive* OR precautions-adoption-process* OR integrated-change* OR i- change* OR theor*) N2 (change* OR modif* OR alter* OR model* OR framework* OR intervention* OR theor*)) OR fishbein OR ajzen OR prochaska OR diclemente OR hochbaum OR kegels OR rosenstock OR bandura OR Weinstein OR de-vries) OR **AB** (((behavior* OR behaviour* OR habit* OR practice* OR attitude* OR ASE OR trans-theor* OR health-belief* OR social-cognitive* OR precautions-adoption-process* OR integrated-change* OR i-change* OR theor*) N2 (change* OR modif* OR alter* OR model* OR framework* OR intervention* OR theor*)) OR fishbein OR ajzen OR prochaska OR diclemente OR hochbaum OR kegels OR rosenstock OR bandura OR Weinstein OR de-vries)**) AND (TI** (immunocompromi* OR compromi* OR immunosuppress* OR autoimmune* OR auto-immune* OR arthritis* OR rheuma* OR psoriasis* OR IBD OR SLE OR lupus* OR systemic-scleros* OR inflammatory- bowel* OR crohn* OR colitis* OR ((HIV OR immunodeficiency OR cancer*) N2 (patient* OR adult* OR survivor* OR person*)) OR ((transplant* OR graft*) N2 (recipient* OR host* OR stem-cell* OR organ*))) OR **AB** (immunocompromi* OR compromi* OR immunosuppress* OR autoimmune* OR auto-immune* OR arthritis* OR rheuma* OR psoriasis* OR IBD OR SLE OR lupus* OR systemic-scleros* OR inflammatory-bowel* OR crohn* OR colitis* OR ((HIV OR immunodeficiency OR cancer*) N2 (patient* OR adult* OR survivor* OR person*)) OR ((transplant* OR graft*) N2 (recipient* OR host* OR stem-cell* OR organ*)))**) OR ((TI** (((vaccin* OR immuniz* OR

immunis*) N2 (cover* OR uptake* OR adherence* OR compliance* OR hesitan* OR behavior* OR behaviour* OR decision* OR status* OR rate* OR acceptanc* OR refus*) N2 (clinician* OR nurs* OR physician* OR specialist* OR doctor* OR personnel OR provider* OR health-care-worker* OR HCWs OR HCW OR professional* OR practitioner*))) OR **AB** (((vaccin* OR immuniz* OR immunis*) N2 (cover* OR uptake* OR adherence* OR compliance* OR hesitan* OR

behavior* OR behaviour* OR decision* OR status* OR rate* OR acceptanc* OR refus*) N2 (clinician* OR nurs* OR physician* OR specialist* OR doctor* OR personnel OR provider* OR health-care-worker* OR HCWs OR HCW OR professional* OR practitioner*)))**) AND (TI** (((behavior* OR behaviour* OR ASE OR trans-theor* OR health-belief* OR social-cognitive* OR precautions-adoption-process* OR integrated- change* OR i-change* OR theor*) N2 (change* OR modif* OR alter* OR model* OR framework* OR intervention* OR theor*)) OR fishbein OR ajzen OR prochaska OR diclemente OR hochbaum OR kegels OR rosenstock OR bandura OR Weinstein OR de-vries) OR **AB** (((behavior* OR behaviour* OR ASE OR trans-theor* OR health-belief* OR social-cognitive* OR precautions-adoption-process* OR integrated- change* OR i-change* OR theor*) N2 (change* OR modif* OR alter* OR model* OR framework* OR intervention* OR theor*)) OR fishbein OR ajzen OR prochaska OR diclemente OR hochbaum OR kegels OR rosenstock OR bandura OR Weinstein OR de-vries)**) OR ((TI** (((vaccin* OR immuniz* OR immunis* OR guideline*) N2 (cover* OR uptake* OR adherence* OR compliance* OR hesitan* OR behavior* OR behaviour* OR decision* OR status* OR rate* OR acceptanc* OR refus*))) OR **AB** (((vaccin* OR immuniz* OR immunis* OR guideline*) N2 (cover* OR uptake* OR adherence* OR compliance* OR hesitan* OR behavior* OR behaviour* OR decision* OR status* OR rate* OR acceptanc* OR refus*))))**) AND (TI** (((behavior* OR behaviour* OR habit* OR practice* OR attitude* OR ASE OR trans-theor* OR health-belief* OR social-cognitive* OR precautions-adoption-process* OR integrated-change* OR i- change* OR theor*) N2 (change* OR modif* OR alter* OR model* OR framework* OR intervention* OR theor*)) OR fishbein OR ajzen OR prochaska OR diclemente OR hochbaum OR kegels OR rosenstock OR bandura OR Weinstein OR de-vries) OR **AB** (((behavior* OR behaviour* OR habit* OR practice* OR attitude* OR ASE OR trans-theor* OR health-belief* OR social-cognitive* OR precautions-adoption- process* OR integrated-change* OR i-change* OR theor*) N2 (change* OR modif* OR alter* OR model* OR framework* OR intervention* OR theor*)) OR fishbein OR ajzen OR prochaska OR diclemente OR hochbaum OR kegels OR rosenstock OR bandura OR Weinstein OR de-vries)**) AND (TI** (travel* OR VFR OR visiting-friends-and-relatives* OR expat* OR emigrant* OR pilgrim* OR touris*) OR **AB** (travel* OR VFR OR visiting-friends-and-relatives* OR expat* OR emigrant* OR pilgrim* OR touris*)**))**

SocINDEX EBSCO – zonder behavior element (Via Laura Doornekamp / Maastricht University)

((TI (((vaccin* OR immuniz* OR immunis*) N2 (cover* OR uptake* OR adherence* OR compliance* OR hesitan* OR behavior* OR behaviour* OR decision* OR status* OR rate* OR acceptanc* OR refus*))) OR **AB** (((vaccin* OR immuniz* OR immunis*) N2 (cover* OR uptake* OR adherence* OR compliance* OR hesitan* OR behavior* OR behaviour* OR decision* OR status* OR rate* OR acceptanc* OR refus*))))**) AND (TI** (immunocompromi* OR compromi* OR immunosuppress* OR autoimmune* OR auto- immune* OR arthritis* OR rheuma* OR psoriasis* OR IBD OR SLE OR lupus* OR systemic-scleros* OR inflammatory-bowel* OR crohn* OR colitis* OR ((HIV OR immunodeficiency OR cancer*) N2 (patient* OR adult* OR

survivor* OR person*)) OR ((transplant* OR graft*) N2 (recipient* OR host* OR stem-cell* OR organ*))) OR **AB** (immunocompromi* OR compromi* OR immunosuppress* OR autoimmune* OR auto-immune* OR arthritis* OR rheuma* OR psoriasis* OR IBD OR SLE OR lupus* OR systemic-scleros* OR inflammatory-bowel* OR crohn* OR colitis* OR ((HIV OR immunodeficiency OR cancer*) N2 (patient* OR adult* OR survivor* OR person*)) OR ((transplant* OR graft*) N2 (recipient* OR host* OR

stem-cell* OR organ*))))) **OR ((TI** (((vaccin* OR immuniz* OR immunis*) N2 (cover* OR uptake* OR adherence* OR compliance* OR hesitan* OR behavior* OR behaviour* OR decision* OR status* OR rate* OR acceptanc* OR refus*) N2 (clinician* OR nurs* OR physician* OR specialist* OR doctor* OR personnel OR provider* OR health-care-worker* OR HCWs OR HCW OR professional* OR practitioner*))) OR **AB** (((vaccin* OR immuniz* OR immunis*) N2 (cover* OR uptake* OR adherence* OR compliance* OR hesitan* OR behavior* OR behaviour* OR decision* OR status* OR rate* OR acceptanc* OR refus*) N2 (clinician* OR nurs* OR physician* OR specialist* OR doctor* OR personnel OR provider* OR health- care-worker* OR HCWs OR HCW OR professional* OR practitioner*))))) **OR ((TI** (((vaccin* OR immuniz* OR immunis* OR guideline*) N2 (cover* OR uptake* OR adherence* OR compliance* OR hesitan* OR behavior* OR behaviour* OR decision* OR status* OR rate* OR acceptanc* OR refus*))) OR **AB** (((vaccin* OR immuniz* OR immunis* OR guideline*) N2 (cover* OR uptake* OR adherence* OR compliance* OR hesitan* OR behavior* OR behaviour* OR decision* OR status* OR rate* OR acceptanc* OR refus*))))) **AND (TI** (travel* OR VFR OR visiting-friends-and-relatives* OR expat* OR emigrant* OR pilgrim* OR touris*) OR **AB** (travel* OR VFR OR visiting-friends-and-relatives* OR expat* OR emigrant* OR pilgrim* OR touris*)))
