# Supplementary material for: Determinants of Vaccination Uptake in Risk Populations: A Comprehensive Literature Review
Source: Vaccines (Basel). 2020 Aug 27;8(3):480. doi: 10.3390/vaccines8030480 (PMC7563537; doi:10.3390/vaccines8030480)
Supplement: Supplementary file 1 [file vaccines-08-00480-s001.zip › Supplementary Table S1. Health behaviour theories 200823.pdf]

Table S1. Health behaviour theories on the basis of the I-Change model.

Health-behaviour Model (abbreviation)	Author, year	Components or Constructs or Stages (→)	Based on
Health Belief Model (HBM)	Hochbaum, Kegels & Rosenstock, 1952 [1]	• Perceived susceptibility, severity, benefits and barriers<br>• Self-efficacy<br>• Call-to-action	
Theory of Reasoned Action (TRA)	Fishbein, Ajzen, 1967 [2]	• Attitude toward behaviour<br>• Subjective norms<br>• Intention to engage in a certain behavior	
Trans-Theoretical Model (TTM)	Prochaska, DiClemente, 1977 [3]	• Precontemplation →<br>• Contemplation →<br>• Preparation →<br>• Action →<br>• Maintenance →<br>• Termination	
Theory of Planned Behaviour (TPB)	Ajzen, 1985 [4]	• Attitude toward behaviour<br>• Subjective norm<br>• Perceived behaviour control<br>• Intention to behaviour<br>• Behaviour	TRA
Social Cognitive Theory (SCT)	Bandura, 1986 [5]	• Reciprocal Determinism (interaction person-environment-behaviour)<br>• Behavioural Capability<br>• Observational Learning<br>• Reinforcements<br>• Expectations<br>• Self-efficacy	Social Learning Theory (Bandura, 1960)
Precaution Adoption Process Model (PAPM)	Weinstein, 1988 [6]	• Unaware →<br>• Unengaged →<br>• Undecided →	

		• Decided not to act OR<br>• Decided to act →<br>• Acting →<br>• Maintenance	
Attitude - Social Influence - Efficacy model (ASE)	De Vries, Dijkstra, & Kuhlman, 1988 [7]	• Attitude<br>• Social Influence<br>• Self-Efficacy	
Integrated Change Model (I-Change)	De Vries, 2004 [8]	• Predisposing determinants<br>• Awareness determinants<br>• Information determinants<br>• Motivation determinants<br>• Intention<br>• Abilities<br>• Barriers	ASE, TRA, SCT, TTM and PAPM

Abbreviations: HBM = Health Belief Model; TRA = Theory of Reasoned Action; TTM = Trans-Theoretical Model; TPB = Theory of Planned Behaviour; SCT = Social Cognitive Theory; PAPM = Precaution Adoption Process Model; ASE = Attitude Social Influence Efficacy model; I-Change = Integrated Change Model.

References

1. Taylor, D.; Bury, M.; Campling, N.; Carter, S.; Garfied; Newbould, D.J.; Rennie, D.T. *A review of the use of the health belief model (hbm), the theory of reasoned action (tra), the theory of planned behaviour (tpb) and the trans-theoretical model (ttm) to study and predict health related behaviour change*; National Institute for Health and Clinical Excellence: London, UK, 2006.
2. LaCaille, L. Theory of reasoned action. In *Encyclopedia of behavioral medicine*, Gellman, M.D.; Turner, J.R., Eds. Springer New York: New York, NY, 2013; pp 1964–1967.
3. Prochaska, J.O.; Velicer, W.F. The transtheoretical model of health behavior change. *American Journal of Health Promotion* **1997**, *12*, 38–48.
4. Ajzen, I. The theory of planned behavior In *Organizational behavior and human decision processes*, Academic Press. Inc.: 1991; Vol. 50, pp 179-211.
5. Bandura, A. Social cognitive theory. Available online: https://www.sciencedirect.com/topics/immunology-and-microbiology/social-cognitive-theory (accessed on 31 December 2019),
6. Weinstein, N.D.; Sandman, P.M.; Blalock, S.J. The precaution adoption process model. In *Health behavior and health education*, 2008; pp 123–147.

7. de Vries, H.; Dijkstra, M.; Kuhlman, P. Self-efficacy: The third factor besides attitude and subjective norm as a predictor of behavioural intentions. *Health Educat. Res.* **1988**, *3*, 273–282.
8. de Vries, H.; Mudde, A.; Leijs, I.; Charlton, A.; Vartiainen, E.; Buijs, G.; Clemente, M.P.; Storm, H.; Navarro, A.G.; Nebot, M., *et al.* The european smoking prevention framework approach (efsa): An example of integral prevention. *Health Educat. Res.* **2003**, *18*, 611–626.
